# Effect of Specimen Geometry on the Thermal Desorption Spectroscopy Evaluated by Two-Dimensional Diffusion-Trapping Coupled Model

**DOI:** 10.3390/ma14061374

**Published:** 2021-03-12

**Authors:** Yafei Wang, Songyan Hu, Guangxu Cheng

**Affiliations:** School of Chemical Engineering and Technology, Xi’an Jiaotong University, Xi’an 710049, China; hu3118316063@stu.xjtu.edu.cn (S.H.); gxcheng@xjtu.edu.cn (G.C.)

**Keywords:** thermal desorption spectroscopy, hydrogen, diffusion, trapping, simulation

## Abstract

The hydrogen diffusion process in ferritic steel during thermal desorption tests was simulated using the finite element method based on the two-dimensional diffusion-trapping coupled model. This model was first verified by experimental data to obtain a physically meaningful combination of trap/lattice parameters. Then, the effect of specimen geometry was studied by varying the height of cylindrical specimens with other parameters fixed at constant values. Simulation of desorption spectra with different specimen geometries indicates that the measurement of hydrogen concentration is not affected by the change in specimen geometry due to the mass conservation law, for original thermal desorption spectra (TDS), which are, however, unlikely to be detected in traditional experiments due to the necessity of specimen transfer procedures. Considering the hydrogen escape during rest time (specimen preparation/transfer/evacuation), the measured TDS curves are expected to be strongly dependent on the specimen geometry. The effect of specimen geometry on desorption spectra is more pronounced for smaller specimens, resulting in the dramatic decrease in peak flux and the increased error of Kissinger method in the determination of trap deactivation energy. The present study may contribute to better understanding and more reliable interpretation of the TDS curves by considering the size effect.

## 1. Introduction

Hydrogen is one of the most promising clean energy sources due to its features of zero emission and no pollution. However, the safety of hydrogen transportation and storage remains a challenging problem especially for hydrogen gas pressure vessels, since the presence of hydrogen can result in dramatic material degradation and consequently unexpected failures of structures [1,2,3]. Prevention of hydrogen-assisted failures requires in-depth understanding of the hydrogen diffusion process and its interaction with local microstructures and stress fields [4,5]. The hydrogen trapping behavior in materials is thereby an important factor because it slows down the diffusion and affects the level of diffusive hydrogen at lattice sites.

The popular method for precisely measuring the trapping parameters of hydrogen for a specific material is thermal desorption spectroscopy (TDS), during which the hydrogen atoms in trap sites gradually escape with the increase in temperature, diffuse out of the specimen through lattice sites and are detected by the instrument. The features of flux–temperature curves, i.e., the position ($T_{m}$) and height ($J_{m}$) of the local peaks, provide useful information about the hydrogen traps. By conducting multiple tests at different heating rates (ϕ), the most representative trap parameter, i.e., the trap deactivation energy ($E_{d}$), is obtained. $E_{d}$ equals to the difference between diffusion activation energy in lattice sites (Q) and trap binding energy (ΔH). It can be quantitatively estimated from the slope in the $\ln\left(\frac{\phi }{T_{m}^{2}}\right)–1/T_{m}$ plot, referred to as the Kissinger theory [6] or Choo–Lee plot [7], as shown in Equation (1):

(1) $$\frac{d\ln\left(\phi /T_{m}^{2}\right)}{d\left(1/T_{m}\right)}=\frac{E_{d}}{R}=\frac{\Delta H-Q}{R}$$

where *R* is the gas constant. The good linear relationship between $\ln\left(\frac{\phi }{T_{m}^{2}}\right)$ and $1/T_{m}$ has also been reported in multiple papers [8,9,10], indicating the likely validity of Kissinger theory in many cases. To better understand hydrogen transport, reliable modeling of the diffusion and trapping processes is necessary. Most of the numerical models regarding diffusion-trapping coupled simulation are based on the Oriani’s local equilibrium theory [11] and the McNabb-Foster theory [12]. It has been shown that these two models are closely related with each other, but they are expressed in different forms [13]. A detailed description and review of these models can be found in literature [14,15]. The idea of ‘equilibrium’ suggests that the hydrogen atoms at lattice sites and those at trap sites are in equilibrium states. The hydrogen atoms at trap sites can be released if the concentration at lattice sites is decreased, acting as hydrogen sources. If the hydrogen concentration at lattice sites is increased, the trap sites can act as hydrogen sinks. These two cases correspond to hydrogen desorption and hydrogen charging, respectively. The hydrogen concentration at lattice sites is thereby greatly dependent on the characteristics of the trap sites, such as trap density and trap binding energy.

Although the diffusion term is not included in Equation (1), it has been proved by Kirchheim [16] through approximate analytical solutions for pure iron that the Choo–Lee equation is intrinsically consistent with the diffusion-trapping coupled model. Nevertheless, many studies have focused on the evaluation of the validity of Kissinger theory, where the desorption spectra at different heating rates were generated using prescribed trapping parameters and the Kissinger theory was applied to extract the trap parameters from the simulated spectra. Multiple studies report the good linear relationship in the Choo–Lee plots for the reproduced TDS curves by numerical simulation, while the trap binding energy determined from the slope deviates from the prescribed values in simulation depending on the parameters used [13,16,17,18].

Experiments show that the TDS curves are strongly dependent on the test parameters, such as specimen geometry, transfer time, etc., which implies that the TDS measurement and parameter extraction may be affected by these factors in testing procedures. Escobar et al. [19] experimentally demonstrated that both the temperature and height of the local peak decrease with specimen thickness. However, Song et al. [20] showed through numerical simulation that peak flux increases while peak temperature decreases with specimen thickness, which are contradictory to the experimental phenomenon. Moreover, different shapes and sizes of TDS specimens have been reported in literature such as rods [21], disks [8], and block specimens [22]. Most of the models in literature consider the simplified 1D model. Although some studies focus on the 2D models [8,23,24], it has not been fully clarified how the shape of specimen geometry affects the TDS curves and consequently the determination of trapping parameters. In this paper, the asymmetrical 2D diffusion-trapping model was used to simulate the hydrogen desorption processes and the flux–temperature curves were generated under different geometries by varying the height/diameter ratios. The influence of specimen geometry on the desorption spectra was investigated, which can contribute to the better interpretation of experimental TDS curves. This paper is organized as follows. First, the simulation was verified by comparing it with experimental data in literature, from which a physically meaningful combination of trap parameters (trap deactivation energy and trap density) could also be obtained. Second, using these trap parameters, the desorption curves were simulated for different specimen geometries with a 2D finite element model, under two different assumptions, i.e., constant specimen diameter and constant specimen volume. The effects of specimen geometry on the desorption spectra and the validity of Kissinger theory were evaluated.

## 2. Numerical Model

The hydrogen transport process during TDS tests can be described by the diffusion-trapping coupled model based on local equilibrium theory [13], as shown in Equation (2):

(2) $$\frac{\partial \theta_{L}}{\partial t}\left[1+\frac{\alpha N_{T}K}{\beta N_{L}{\left(1+K\theta_{L}\right)}^{2}}\right]+\frac{\alpha N_{T}K\Delta H\phi \theta_{L}}{\beta N_{L}RT^{2}{\left(1+K\theta_{L}\right)}^{2}}=D_{L}\frac{\partial^{2}\theta_{L}}{\partial x^{2}}$$

where $\theta_{L}$ is the occupation fraction of lattice sites, β and α are the number of atoms sites per trap, and $N_{L}$ and $N_{T}$ are the trap densities. $D_{L}$ is the diffusion coefficient of hydrogen at lattice sites and determined by $D_{L}=D_{0}\exp\left(-Q/RT\right)$, given the temperature *T*, lattice activation energy Q, and diffusion pre-exponential factor $D_{0}$. In the desorption tests, the temperature *T* is a function of time: $T=T_{0}+\phi t$, with ϕ as the heating rate. The hydrogen concentration at lattice site $c_{L}$ and trap sites $c_{T}$ can be determined by $c_{L}=\theta_{L}\beta N_{L}$ and $c_{T}=\theta_{T}\alpha N_{T}$, respectively. Oriani’s equilibrium theory assumes the local equilibrium between lattice sites and trap sites [11]. Thereby, the relationship between $\theta_{L}$ and $\theta_{T}$ can be expressed as $\theta_{T}=\frac{K\theta_{L}}{1+K\theta_{L}}$, where the equilibrium constant *K* is determined by the trap binding energy: $K=\exp\left\{\frac{-\Delta H}{RT}\right\}$. Equation (2) describes the fully coupled diffusion and trapping processes for a single trap based on the local equilibrium assumption, which contains various parameters including the lattice parameters $N_{L}$,$\;\theta_{L}^{0}$, $D_{0}$, and Q, trap parameters ΔH and $N_{T}$, and test parameters $T_{0}$ and ϕ. Besides, the temperature gradient in the specimen is usually not considered in the simulation of desorption spectra due to the small size of the specimen, implying that the thermal conduction process is much faster than the diffusion process. Moreover, although the trap sites are related to local microstructural features in materials such as grain boundaries, dislocations, precipitations, etc., the trap sites are usually assumed to be uniformly distributed through the whole specimen in the simulation of desorption spectra.

**Figure 1:**
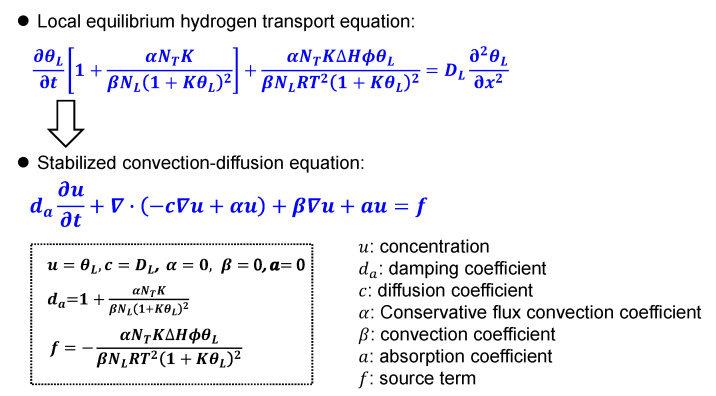
Analogy of the hydrogen transport equation to the stabilized convection–diffusion equation in COMSOL software.

Equation (2) was solved using the COMSOL Multiphysics (Burlington, VT, US) software by analogy to the stabilized convection–diffusion model as shown in Figure 1.

The partial differential equation becomes nonlinear when the coefficients are dependent on the variable *u* [25]. The convection term is not included in the simulation. As a result, the physical governing equation corresponds to a diffusion problem, with the hydrogen trapping effect acting as the source term uniformly distributed throughout the specimen. In the two-dimensional symmetric model, the rod and disk-shaped specimens were considered with the diameter fixed at 4 mm and height varied from 0.4 to 40 mm, corresponding to the ratios of height/diameter of 0.1 to 10. The parameters used in the analysis were: $N_{L}=8.46\times 10^{28}$ atoms/m^3^, α=β=1, $T_{0}$ = 293 K [13]. The initial occupation fraction of hydrogen at lattice site $\theta_{L}^{0}$ was fixed at $3\times 10^{-7}$. The total flux was calculated by summing all the fluxes at the external surfaces. The mesh number along the smallest length of specimen was 40 and the time increment was 2 s, which were proved to be sufficient to obtain mesh-independent results. Other boundary conditions were set to fully simulate the actual conditions in TDS tests, which have been discussed in detail in our previous study [17]. The typical experimental procedure for TDS tests is as follows. The specimens were charged with hydrogen, then the surfaces of specimens were cleaned by water or alcohol, after cleaning by abrasive paper. Then, the specimens were transferred and placed into the chamber of TDS instrument. The air in the chamber was evacuated to build a high vacuum environment. Finally, the temperature was elevated, during which the release of hydrogen atoms was measured by the detector. A considerable amount of hydrogen atoms can be lost during these steps. Therefore, another important parameter is the rest time, $t_{r}$, i.e., the time from the end of hydrogen charging to the start of data collecting, which can vary significantly depending on the testing method, instrument, and specimen preparation procedures.

## 3. Results and Discussion

### 3.1. Validation with Experiment

**Figure 2:**
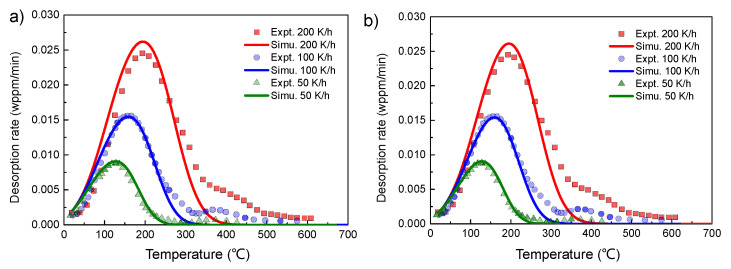
Comparison of the simulated thermal desorption spectra (TDS) curves with the experimental data reported in Reference [22], using two different values of rest time: (**a**) $t_{r}$ = 3 h and (**b**) $t_{r}$ = 5 h. Other parameters used in the simulation were: $E_{d}=Q-\Delta H=-25\;\mathrm{kJ}/\mathrm{mol}$, Q=−4.15 kJ/mol, $D_{0}=5.1\times 10^{-8}\;m^{2}/s$. The rod specimen was considered with a diameter *D* = 6.3 mm and height *H* = 25 mm. By finding a best fit with the experimental data, different combinations of $N_{T}/N_{L}=8.0\times 10^{-2}$, $\theta_{L}^{0}=3.0\times 10^{-7}$ and $N_{T}/N_{L}=7.5\times 10^{-2}$, $\theta_{L}^{0}=3.6\times 10^{-7}$ were used in (**a**,**b**), respectively.

First of all, the numerical model was verified by comparing with experimental data in literature. There are many studies in literature involving the TDS tests using different specimens. However, these studies involve different materials with a wide range of trap parameters. Verification of the model by comparing with the experimental data for different geometries in one paper is somewhat difficult, due to undetermined trap binding energy in the experiment. Note that good fitting can be achieved for a single TDS curve by using different combinations of trap parameters and lattice parameters. Instead, the simulation was compared with multiple desorption spectra at different heating rates, to obtain a physically meaningful combination of trap/lattice parameters, as shown in Figure 2.

The experimental TDS curves were obtained at three heating rates, i.e., 50, 100, and 200 K/h, for 4130X steel after thermal charging in 100 MPa H_2_ environment at 85 °C for 210 h [22]. In the above study, block specimens were used with a size of 25 × 13 × 6.3 mm^3^. For the consideration of computation efficiency, the two-dimensional symmetrical geometry with a diameter *D* = 6.3 mm and height *H* = 25 mm was considered in the simulation, instead of the 3D geometry utilized in the experiment. As will be shown shortly, the decrease in the specimen size has some influence on the desorption spectra, resulting in the negative shift of peak temperature and peak flux. Therefore, the estimated trap activation and trap density are expected to be slightly lower than the ‘real’ ones estimated from the 3D model, although the true values are difficult to obtain due to other uncertainties. As can be seen in Figure 2, a prominent peak was observed in the temperature range of 125–190 °C, with slight overlapping with the insignificant high-temperature peak. The trap deactivation energy determined from the Choo–Lee method in the above work was ~25 kJ/mol. Using this value, the TDS curves were simulated by finding the best fit with the experimental data, by varying the trap density $N_{T}$ and initial occupation fraction of hydrogen at lattice site $\theta_{L}^{0}$, with other parameters fixed as follows:$\;E_{d}=-25\;\mathrm{kJ}/\mathrm{mol}$, Q=−4.15 kJ/mol, and $D_{0}=5.1\times 10^{-8}\;m^{2}/s$. A good agreement was found between the simulated and experimental curves when $N_{T}/N_{L}=8.0\times 10^{-2}$ and $\theta_{L}^{0}=3.0\times 10^{-7}$. Both the peak temperature and peak flux match well with the experiment. However, a slight deviation is observed for the peak width, which is partially attributed to the influence of the high temperature peak. Note that the rest time was not given in the experiment, thus the accurate determination of the trap parameters is difficult. Since the rest time can greatly affect the desorption rate at the starting temperature, the results in Figure 2 imply that the rest time used in simulation should be close to the real one, as indicated by the good consistency at the starting point. The inaccurate determination of rest time will cause some deviation of the values of $N_{T}$ and $\theta_{L}^{0}$. By comparing Figure 2a,b it can be seen that, with the increase in rest time, the decrease of $N_{T}$ and increase of $\theta_{L}^{0}$ have to be modified to match the experimental data. Additionally, the second peak in the experimental data indicates the presence of a different kind of trap site. However, the simulation of TDS curves with multiple kinds of traps remains challenging in some cases due to the overlapping of local peaks [26], which still needs further investigation. Nonetheless, the comparison of simulation and experimental results not only validates the numerical model here, but also helps to simplify the analysis in the present study by fixing some of the influencing factors. In the following analysis, the lattice parameters and trap parameters were the same as those used in Figure 2a, while the geometries were varied to study the influence on desorption spectra.

### 3.2. Effect of Specimen Geometry

#### 3.2.1. Effect on TDS Curves

The effect of specimen geometry on the TDS curves was studied by changing the ratio of specimen height/diameter from 0.1 to 10, using the same parameters in Figure 2. To better control the mesh number, the diameter was fixed at 4 mm instead of the value reported in experiment, i.e., 6.3 mm. The spectra with different combinations of *H* and *D* were simulated, among which the combination of *H* = 20 mm and *D* = 4 mm was the closest to the experimental condition.

**Figure 3:**
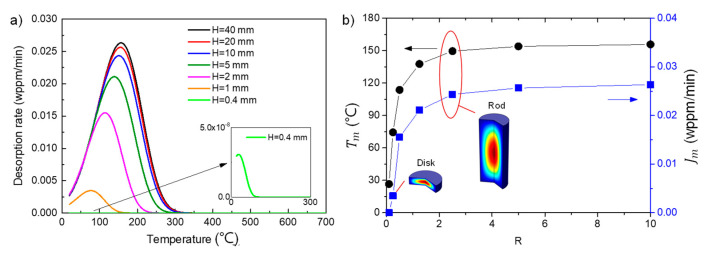
(**a**) Effect of the ratio of specimen height/diameter (*R* = *H*/*D*) on desorption spectra with the diameter fixed at 4 mm and height decreased from 40 to 0.4 mm and (**b**) the variation of peak temperature $T_{m}$ and peak flux $J_{m}$ with *R*. The parameters used in the simulation were identical with those in Figure 2, except that the heating rate was fixed at 200 K/h. Note that the rest time was $t_{r}$ = 3 h.

It is observed from Figure 3 that both peak temperature and peak flux decrease apparently with the decrease in specimen height, which agrees with the experimental observations reported by Escobar et al. [19].

For the rod specimens, the influence of specimen geometry on the desorption spectra is small if the specimen height reaches a sufficiently large value, e.g., H > 10 mm. This is because the flux for the long-rod specimens is mainly composed of the fluxes at the side surfaces, while the fluxes at the top and bottom surfaces only occupy a small fraction. In this way, the 2D cylindrical problem is similar with the 1D problem except that the axial coordinates are used in the 2D model. However, a dramatic decrease of $T_{m}$ and $J_{m}$ is observed when the specimen changes from rod specimen to block specimen, i.e., the specimen height becomes similar with the diameter (*R* = 1). This is because the hydrogen escapes faster along the axial direction (the amount of hydrogen lost per unit time is higher) than the radial direction due to the shorter distance from the center of specimen to the surface and the fluxes at the top/bottom surfaces dominate the total flux. For a sufficiently small specimen height, i.e., H = 0.4 mm, the flux can be barely detected. This is attributed to the significant amount of hydrogen lost during the rest time, i.e., $t_{r}$ = 3 h.

**Figure 4:**
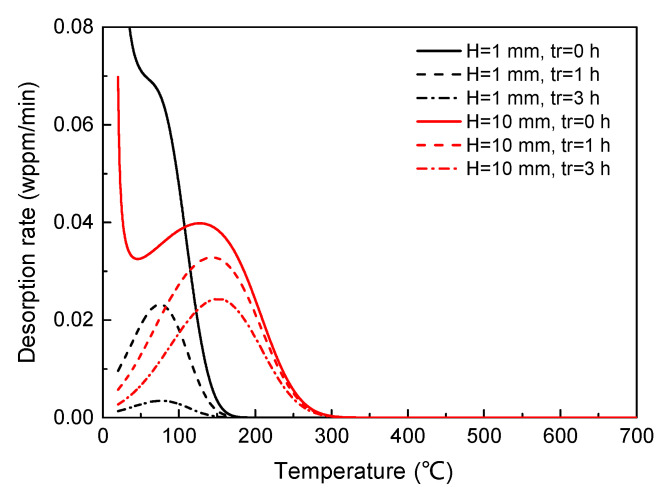
Simulated desorption spectra with different specimen geometries (H = 1, 10 mm) and rest time ($t_{r}\;$ = 0, 1, 3 h), showing the faster escape of hydrogen for smaller specimen. Both peak temperature and peak flux are strongly affected due to the change in specimen geometry. The heating rate was fixed at 200 K/h.

To rule out the influence of hydrogen loss, the dependence of the original TDS curves (rest time is zero) on specimen geometry is presented in Figure 4.

It is seen that the peak temperature shifts negatively while the peak flux increases apparently with the decrease in specimen height, while the total hydrogen concentration remains constant. However, the hydrogen loss is much faster in the smaller specimen (H = 1 mm) than the larger specimen (H = 10 mm), as indicated by the significant decrease in flux from $t_{r}$ = 0 h to 3 h for H = 1 mm. Since air exposure is inevitable during experiment, it is expected that the experimentally observed peak flux will decrease dramatically with the decrease in specimen geometry despite the opposite trend for the original curves (which is undetectable in practical experiment). In other words, the measurement of total hydrogen concentration is more sensitive to the specimen geometry and testing procedures when the size is small.

**Figure 5:**
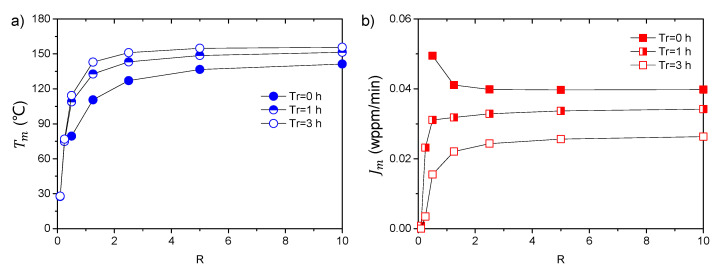
Effect of specimen geometry and rest time on (**a**) the peak temperature and (**b**) the peak flux, using identical parameters to those in Figure 3. For the original desorption spectra with $t_{r}$ = 0 h, the peak temperature decreases but the peak flux increases with specimen dimension.

For clarity, the variations of $T_{m}$ and $J_{m}$ with *R* for different rest times are shown in Figure 5.

Consistent with the results in Figure 4, the peak temperature $T_{m}$ decreases gradually with the decrease in *R* for all rest times. The variation of peak flux $J_{m}$, however, exhibits clear dependence on the rest time, indicating dramatic flux decrease for small specimens. This may affect the TDS measurement in several aspects: firstly, the measured hydrogen concentration is strongly dependent on the specimen geometry and it will become significantly small or even undetectable by the instrument for the small disk-shaped specimen, due to the fast escape of hydrogen along thickness direction; secondly, the determination of hydrogen concentration will be more sensitive to the experimental procedures for small specimens, especially the aging time, which can probably result in the poor reproducibility of data since it is difficult to control and record the exact rest time from the ending of hydrogen charging to the start of data collection; and thirdly, the shift of peak temperature may affect the determination of trap parameters, i.e., the validity of Kissinger theory.

#### 3.2.2. Validity of Kissinger Theory

It is expected that the specimen geometry would affect not only the measurement of hydrogen concentration but also the determination of trap parameters. Since the validity of Kissinger theory is probably dependent on trap parameters themselves, as addressed by Raina et al. [13], only the ‘effective’ combination of trap density $N_{T}$ and trap deactivation energy $E_{d}$, which was verified with experimental data in Figure 2, is considered here. The values of $N_{T}$ and $E_{d}$ used in simulation are defined as the ‘true’ values, while those estimated from the simulated TDS curves using Kissinger theory as shown in Equation (1) are defined as the ‘predicted’ values. The comparison of true and predicted values can help evaluate whether or not the Kissinger theory holds for different specimen geometries.

**Figure 6:**
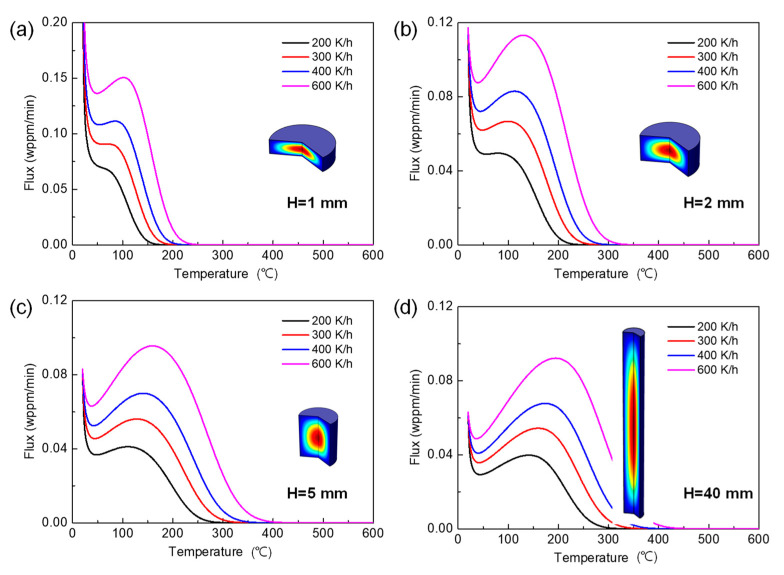
Desorption spectra for different specimen geometries at four different heating rates from 200 to 600 K/h, with the illustration of specimen geometries shown in the inset figures: (**a**) H = 1 mm, (**b**) H = 2 mm, (**c**) H = 5 mm, (**d**) H = 40 mm. The diameter of specimen was fixed at *D* = 4 mm (inset figures shown in different scales). Other parameters are same as those in Figure 2.

The simulated desorption spectra for different specimen geometries at four heating rates from 200 to 600 K/h are shown in Figure 6, with the cylindrical specimens illustrated in the inset figures.

**Figure 7:**
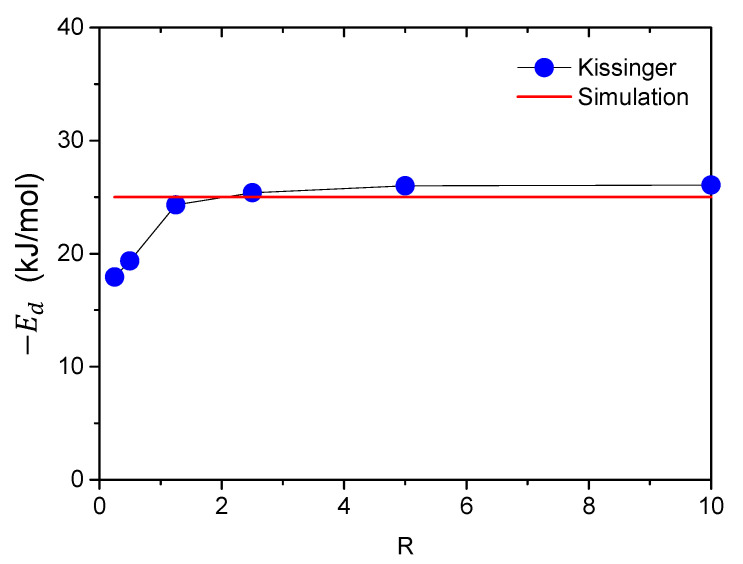
Comparison with the estimated trap deactivation energies *E*_d_ using Kissinger theory (**blue dots**) and those prescribed in the simulation (**red line**) for different *R* values. Four different heating rates of ϕ=200, 300, 400, and 600 K/h were used to generate the Choo–Lee plots.

There is an apparent increase in peak flux when the geometry changes from rod to disk specimens, while the shape of desorption spectra is not affected by the change in specimen geometry. The trap deactivation energy can be determined from the slope of the Choo–Lee plot by Equation (1), as discussed in previous sections. The predicted values of $E_{d}$ using the Kissinger equation are shown in Figure 7 using a prescribed value of $E_{d}\;$ = 25 kJ/mol for the simulated TDS curves.

It is seen that the deviation of the predicted values from the true values is fairly small for large R values, indicating the validity of Kissinger theory. It was verified through simulation that further increase in the mesh number along the radial direction does not apparently improve the accuracy. The minor deviation is probably due to the error of Kissinger theory instead of the finite element model. According to Kirchheim [16], the governing equation of local equilibrium theory can be expressed in the form of Kissinger equation by analytical approximation as Equation (3):

(3) $$\ln\frac{\phi }{T_{m}^{2}}=\frac{E_{d}}{RT_{m}}+\ln\frac{4D_{0}R\pi^{2}\left(N_{L}+N_{T}\right)}{L_{c}^{2}N_{T}(-E_{d})}$$

where $L_{c}$ is the length of the specimen. Equation (3) indicates that the diffusion-trapping coupled local equilibrium equation is intrinsically consistent with the Kissinger equation. However, this approximation assumes that $\frac{\left|\Delta H\right|}{RT_{m}}\gg 1$, which is valid for deep traps but may yield some errors for shallow traps.

**Figure 8:**
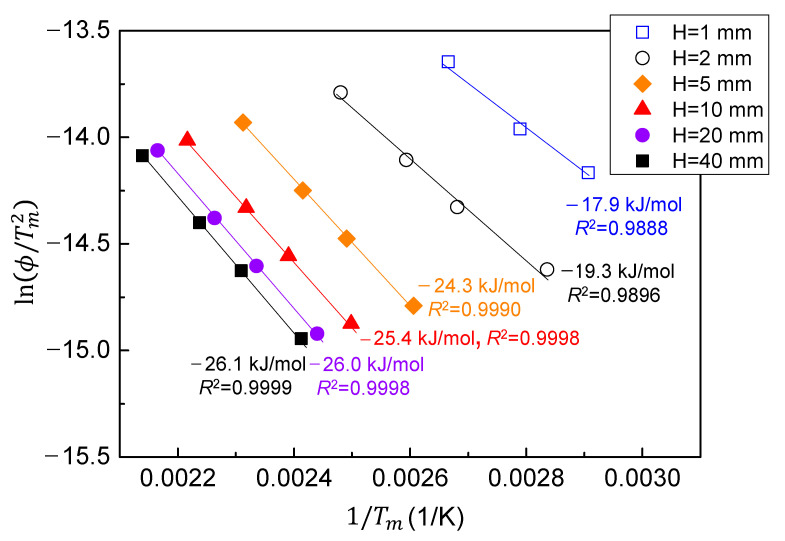
Choo–Lee plot for different specimen geometries (*H* = 1, 2, 5, 10, 20, 40 mm), showing the increase in deactivation energy with the decrease in *H*. Notably, no local peak was identified for *H* = 1 mm at a heating rate of 200 K/h.

The error of Kissinger theory increases dramatically with the decrease in specimen geometry for small disk-shaped specimens. The Choo–Lee plots for different specimen geometries and the parameters are shown in Figure 8 and Table 1.

The estimated trap deactivation energy increases with the decrease in specimen size, indicated by the change in slope of the linear fitting. For a large specimen height, a very good linear fitting can be obtained in the Choo–Lee plot. However, the goodness of linear fitting, as indicated by the values of *R*^2^, decreases with specimen geometry, which implies the increased error of Kissinger theory. The intercept of the linear fitting also shifts apparently with the decrease in specimen size, which can be explained from the analytical approximation shown in Equation (3), indicating dependence of the intercept (second term on the right) on specimen length.

#### 3.2.3. Varied Geometries with Constant Volume

The previous results show the effect of specimen geometry on desorption spectra, with the specimen diameter fixed at constant value while the specimen volume is varied greatly. In this section, the desorption spectra were simulated by changing both the diameter and height, with the specimen volume fixed at constant value.

**Figure 9:**
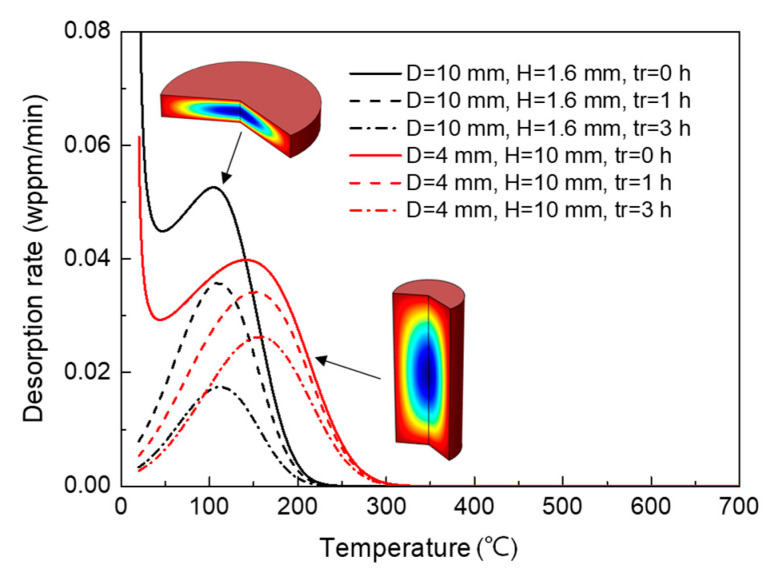
Desorption spectra for different specimen geometries (*D* = 10 mm, *H* = 1.6 mm and *D* = 4 mm, *H* = 10 mm) and rest time. The specimen volume was held constant. Inset figures show the specimen geometries correspondingly.

The simulated desorption spectra for two typical specimen geometries are shown in Figure 9 with the inset figures illustrating the corresponding geometries. It is seen that with the transition from rod specimen to disk-shaped specimen, the peak flux increases apparently while the peak temperature decreases. It is also observed that the hydrogen release becomes faster during specimen transfer, as indicated by the more dramatic decrease in peak flux for disk-shaped specimen. This influence is similar to that of reducing specimen volume for constant diameter, as discussed in the previous section.

**Figure 10:**
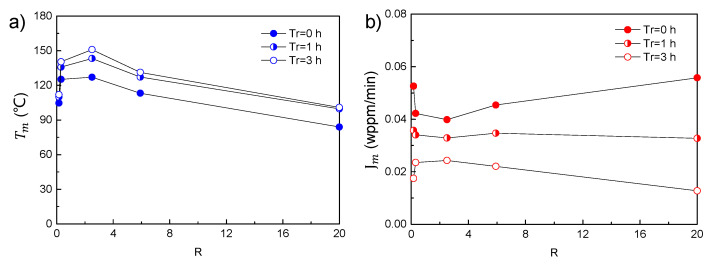
Effect of specimen geometry and rest time on (**a**) the peak temperature and (**b**) the peak flux, using identical parameters as those in Figure 3. The specimen volume was controlled at constant values, while the ratio of specimen height/diameter (*R*) was varied.

Further investigation on the effect of the specimen ratio on $T_{m}$ and $J_{m}$ shows that in the case of constant volume, the effect of specimen geometry is mainly controlled by the characteristic length, as shown in Figure 10. For very small *R* values (*H* < *D*), the characteristic length (*H*) is small, resulting in faster hydrogen release. Accordingly, decreased hydrogen concentration is measured after a considerable period of rest time. This phenomenon is similar for very large *R* values (*H* > *D*), since the characteristic length (*D*) becomes small too. The hydrogen loss along the characteristic length increases sharply in these cases, which results in the dramatic decrease of $J_{m}$ and the negative shift of $T_{m}$. It is reasonably expected that this influence also results in the poor validity of Kissinger theory, which is not shown here.

## 4. Conclusions

The effect of specimen geometry on the measurement of TDS curves and the determination of trap parameters using Kissinger theory was studied using a two-dimensional finite element model based on the Oriani’s local equilibrium theory, taking ferritic steel as an example. The diffusion-trapping coupled partial differential equation was solved by analogy to the stabilized convection–diffusion model in COMSOL software. The numerical model was first verified by the experimental data from literature to determine the ‘effective’ combination of trap/lattice parameters. Then the geometry parameters were varied with other parameters fixed at meaningful values. The simulation indicates significant influence of specimen geometry on the desorption spectra, indicated by the change in peak temperature and peak flux with the variation of specimen size. When the specimen size reaches a sufficiently small value, it would be difficult to detect hydrogen flux in practical experiments due to the significant hydrogen loss during specimen transfer. Although the mass conservation law holds for different specimen geometries, it is expected that the measured hydrogen concentration is dependent on the specimen size, especially for the small disk-shaped specimens. This is due to the dependence of hydrogen loss on specimen thickness. Regarding the trap energy, the Kissinger theory holds well for long rod-shaped specimens, while the error of Kissinger theory increases sharply as the specimen changes from rod to disk specimens. The specimen geometry affects both the slope and intercept of the Choo–Lee plots, which greatly influences the determination of trap energy. In both diameter-constant and volume-constant cases, the effect of geometry on spectra is mainly controlled by the hydrogen loss during specimen transfer. For diameter-constant cases, the hydrogen loss is mainly dependent on the change in specimen volume, while for volume-constant cases, the hydrogen loss mainly depends on the characteristic length. Therefore, it is recommended to use specimens with sufficiently large volume and characteristic length to prevent hydrogen loss during rest time, and to obtain more reliable information on the hydrogen trapping behavior through TDS tests.

## Figures and Tables

**Table 1:** Results of the linear fitting for the Choo–Lee plot for different specimen geometries.

*H*, mm	1	2	5	10	20	40
*A* *	−2155.2	−2327.2	−2925.0	−3053.2	−3127.1	−3134.7
*B* *	−7.9	−8.0	−7.2	−7.2	−7.3	−7.4
*R* ^2^	0.9888	0.9896	0.9990	0.9998	0.9998	0.9999
*E*_d_, kJ/mol	−17.9	−19.3	−24.3	−25.4	−26.0	−26.1
Error, %	−28.4	−22.8	−2.8	1.6	4	4.4

* Coefficients for the linear fitting: $\ln\frac{\phi }{T_{m}^{2}}=\frac{A}{T_{m}}+B$.

## Data Availability

The data presented in this study are available on request from the corresponding author.
